# Invertebrate Metacommunity Structure and Dynamics in an Andean Glacial Stream Network Facing Climate Change

**DOI:** 10.1371/journal.pone.0136793

**Published:** 2015-08-26

**Authors:** Sophie Cauvy-Fraunié, Rodrigo Espinosa, Patricio Andino, Dean Jacobsen, Olivier Dangles

**Affiliations:** 1 IRD, Institut de Recherche pour le Développement, UMR EGCE, IRD-247 CNRS-UP Sud-9191, 91198 Gif-sur-Yvette cedex, France; 2 Pontificia Universidad Católica del Ecuador, Facultad de Ciencias Exactas y Naturales, Quito, Ecuador; 3 Instituto de Ecología, Universidad Mayor San Andrés, Cotacota, La Paz, Bolivia; 4 Freshwater Biological Laboratory, Department of Biology, University of Copenhagen, Universitetsparken 4, 2100 Copenhagen, Denmark; University of Waikato (National Institute of Water and Atmospheric Research), NEW ZEALAND

## Abstract

Under the ongoing climate change, understanding the mechanisms structuring the spatial distribution of aquatic species in glacial stream networks is of critical importance to predict the response of aquatic biodiversity in the face of glacier melting. In this study, we propose to use metacommunity theory as a conceptual framework to better understand how river network structure influences the spatial organization of aquatic communities in glacierized catchments. At 51 stream sites in an Andean glacierized catchment (Ecuador), we sampled benthic macroinvertebrates, measured physico-chemical and food resource conditions, and calculated geographical, altitudinal and glaciality distances among all sites. Using partial redundancy analysis, we partitioned community variation to evaluate the relative strength of environmental conditions (e.g., glaciality, food resource) vs. spatial processes (e.g., overland, watercourse, and downstream directional dispersal) in organizing the aquatic metacommunity. Results revealed that both environmental and spatial variables significantly explained community variation among sites. Among all environmental variables, the glacial influence component best explained community variation. Overland spatial variables based on geographical and altitudinal distances significantly affected community variation. Watercourse spatial variables based on glaciality distances had a unique significant effect on community variation. Within alpine catchment, glacial meltwater affects macroinvertebrate metacommunity structure in many ways. Indeed, the harsh environmental conditions characterizing glacial influence not only constitute the primary environmental filter but also, limit water-borne macroinvertebrate dispersal. Therefore, glacier runoff acts as an aquatic dispersal barrier, isolating species in headwater streams, and preventing non-adapted species to colonize throughout the entire stream network. Under a scenario of glacier runoff decrease, we expect a reduction in both environmental filtering and dispersal limitation, inducing a taxonomic homogenization of the aquatic fauna in glacierized catchments as well as the extinction of specialized species in headwater groundwater and glacier-fed streams, and consequently an irreversible reduction in regional diversity.

## Introduction

One impact of climate change is the acceleration of glacial shrinkage [1, 2], resulting in an alteration of glacial meltwater contribution to alpine stream flow [3, 4]. Although glacier runoff modification depends on glacier size, elevation, catchment characteristics, and the rate of glacier retreat [5], the overall reduction in ice volume is expected to yield a significant increase in annual glacier runoff [6], followed by a decrease until the complete disappearance of the glacier [7, 8]. As alpine glacierized catchments are unique freshwater ecosystems harboring specific species assemblages linked to the dynamics of water source contributions [9, 10], any changes in glacier runoff are likely to affect aquatic biodiversity [11–13]. While previous studies performed spatial sampling at the catchment scale (e.g., [14, 15]), most did not use available spatial analyses that allow identifying physical and ecological processes affecting the spatial distribution of aquatic communities at the catchment scale. Even though the number of aquatic metacommunity studies has considerably increased over the last decades, in particular in running waters [16], to our knowledge, metacommunity theory has never been used as a central concept to assess the mechanisms structuring aquatic species distribution in glacier-fed aquatic ecosystems.

Here, we propose using metacommunity theory to describe spatial patterns of community organization at the catchment scale and identify underlying physical and ecological processes resulting in such patterns. A metacommunity is a set of local communities connected through the dispersal of multiple potentially interacting species [17]. Leibold et al. [17] have provided the basic background and theory of metacommunity structure (pattern of spatial species distribution) and dynamics (mechanisms that arise within metacommunities). Metacommunity theory recognizes two types of forces that can affect the structure of communities: local forces including species interactions and local environmental conditions, and regional forces including dispersal processes of organisms between local communities [18–20]. In particular, Heino et al. [16] proposed that species sorting model (i.e. species are sorted along environmental gradient) generally prevails in aquatic systems, while dispersal limitation varies among different aquatic systems, organisms, and spatial scales.

Stream networks possess several features that differentiate them from the majority of metacommunity configurations [21, 22]. Streams are dendritic ecological networks [23, 24], i.e. systems with a hierarchical branching structure in which mainstems connect multiple branches. The flux of aquatic organisms with low aerial dispersal capacity is predicted to be greater through mainstems relative to headwaters. Indeed, mainstems integrate movement of organisms from and between branches [25] while the more isolated headwater streams only receive aquatic migrants from downstream reaches due to the lack of aquatic colonist source upstream [16]. The directionality of water flow engenders a stronger influence of upstream sites on downstream sites than vice versa due to directional fluxes of matter and energy [21]. Thus, both stream geometry and flow directionality strongly affect organism dispersal [23, 26]. Moreover, depending on the organism, dispersal can occur across watershed (overland dispersal, e.g. adult flying insects; [27]) and/or along the stream channel (watercourse, e.g., diatoms, insect larvae; [28]), either through active behavior (flight, swim, crawling, e.g. upstream movement by fish; [21]) or through passive transport (through animal vectors, by wind or flood, e.g., crustacean, snails, aquatic mites; [29, 30]). In addition, stream networks are highly heterogeneous systems, presenting strong differences in instream environmental conditions among sites, especially in headwater catchments [31], but probably even more in headwater glacierized catchments. Indeed, glacierized catchments exhibit a wide range of streams with contributions from different water sources, from meltwater- to groundwater-dominated streams, thereby creating a high spatial heterogeneity of environmental conditions [32]. Therefore, glacial stream networks likely present particular and complex metacommunity dynamics [26], yet very little information exist about this.

Here, we examined the benthic macroinvertebrate spatial distribution in an Andean glacierized watershed providing a wide gradient of glacial influences to explain, and eventually predict, temporal change that would occur along a glacier melting process. Our study uses metacommunity theory to 1) identify mechanisms driving the organization of the macroinvertebrate metacommunity, in particular the relative influence of local (environmental conditions) vs. regional processes (dispersal), and 2) determine the effect of glacial meltwater contribution to alpine streams flow on the macroinvertebrate community variation among stream sites, either by generating high environmental heterogeneity, or by limiting macroinvertebrate dispersal. We hypothesize that the environmental harshness of glacial meltwater (i.e. low temperature, conductivity, channel stability, and high turbidity; [33]) 1) was the main environmental filter, and 2) affects water-borne dispersal within the stream network. We also examined whether 1) the macroinvertebrate dispersal abilities (flying, non-flying adults) and 2) the community geographical location (first-order vs. mainstems sites) affect our predictions. Finally, we propose a conceptual diagram of the response of alpine macroinvertebrate diversity in the face of glacier melting.

## Material and Methods

### Study site

**Fig 1:**
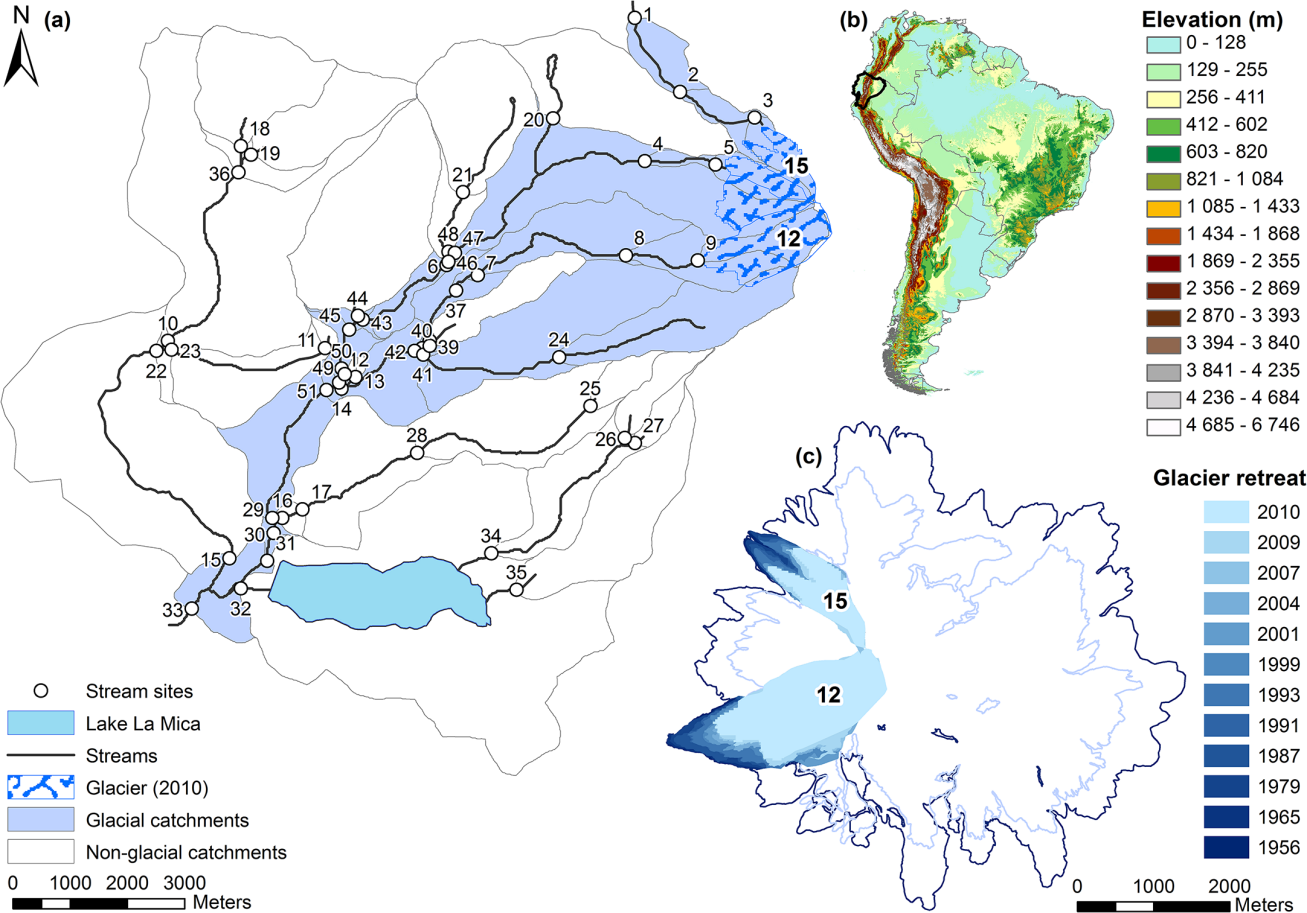
Study area: The Antisana Volcano (Ecuador). (a) Map of the study area at the Antisana volcano. Study sites are represented by open circles. Catchment basins of all sites are represented by polygons; light blue polygons for glacial catchments and white for non-glacial catchments. The catchments were delimited based on a 40m resolution DEM using SAGA GIS (2.0.8). The glacier outlines were computed based on Landsat satellite images from 2010 (see [35] for glacier outlines and catchment delimitation details). (b) Elevation map of South America. South America countries were delimited in grey, with Ecuador highlighted in black. The 30 arc-second digital elevation model was taken from http://www.arcgis.com/features/. (c) Map of the retreat of Antisana’s glaciers since 1956. Total glacier outlines were represented for the years 1956 and 2009. Outlines of glaciers “12” and “15” were represented for 12 years between 1956 and 2010. Glacier outlines were computed from Landsat satellite images. Maps were made using ArcGis (10.0).

The study was conducted in 51 stream sites, located in a 115 km^2^ watershed in the Ecological Reserve of Antisana, Ecuador (0° 33′ 09″S, 78° 14′ 58″W, mouth coordinates; Fig 1B). The watershed was composed of stream catchments showing different glacier influence (Fig 1A). Among the 51 sites, 21 had no glacier influence and 30 were located along four glacier-fed streams and presented between 1 and 93% of glacier cover in the catchment (this percentage was calculated by dividing the glacier area by the total catchment basin area). The four glacier-fed streams originated from the snout of the Antisana glaciers above 4800 m a.s.l. At the time of the study in 2010, glaciers “12” and “15” (the most studied, see Fig 1A for location) covered an area of about 1.82 and 0.60 km^2^, respectively. They lost around 33% of their surface area between 1979 and 2007 [1]. Their average deficit was estimated at 251, 146, and 600 kg m^-2^ yr^-1^ during the 1956–1965, 1965–1993, and 1993–1998 periods, respectively (Fig 1C, [34]). Among the 51 sites, 25 were located on first-order streams (hereafter considered as headwater streams) and 26 on second and third-order streams. Fourteen first-order sites were fed by groundwater and/or rainfall while the 11 others were fed by glacial meltwater. All study sites were located between 3886 and 4835 m a.s.l. Glacially-influenced sites were located at distances of 15 m—15.2 km from the glacier snouts. Glacial floods occurred almost every day in glacier-fed streams and no stream ran dry during the entire study period. In 2009–2010, the Ecological Reserve of Antisana was a private land. The owner gave us the permission to conduct our study there. No specific permissions were required at the Ecological Reserve of Antisana for stream organisms sampling. Our studies involved neither endangered nor protected species.

### Macroinvertebrate sampling

At each site, we randomly collected five quantitative Surber samples (0.05 m^2^; mesh size 200 μm) from pebble-cobble substratum along a 25 m stretch. With five Surber samples, we detected more than 60% of the potential total richness per site, as calculated with the Jackknife species-richness estimator [36]; using the package *vegan* in R (R Development Core Team 2013, version 3.0.2). Macroinvertebrate samples were collected once between May 2009 and January 2010 in the morning before the daily glacial flood and preserved in the field in 70% ethanol. We assumed that differences in sampling dates did not significantly affect the results of our study, as temporal variability in community composition was significantly lower than spatial variability (see S1 Appendix). Moreover, Andean tropical streams are known to present no seasonal pattern in macroinvertebrates assemblage [37, 38] and phenology [39].

In the laboratory, samples were rinsed through a 200-μm sieve and sorted thoroughly by hand in a standardized manner, without use of magnification. No subsampling was applied. Macroinvertebrates were identified under a microscope at 10× magnification range mostly to family and separated into morphospecies, according to Fernández and Domínguez [40].

### Environmental characterization

A detailed characterization of each site was performed the same day we collected macroinvertebrates. Stream width was measured at 5 transects located every 5 m along the 25 m stretch. Water depth was measured 10 times along the 5 transects. We also measured stream slope (see [37] for details on the method) and quantified the physical stability of the stream based on the channel bottom component of the Pfankuch index [41]. Conductivity (at 25°C), water temperature, and pH were measured with portable meters, model Cond 315i and pH 315i, respectively (WTW, Weilheim, Germany). Water turbidity was measured with a Eutech TN-100 Turbidimeter (Eutech, Nijkerk, The Netherlands). We estimated the food resources available to macroinvertebrates by quantifying epilithic algae in 9 small pebbles randomly collected and benthic organic matter obtained in Surber samples (see [37] for details on the methods).

### Overall modeling framework

To test each of our three hypotheses related to the spatial organization of benthic macroinvertebrate assemblages, we built three comparative models.

Model 1 compared the relative importance of the environmental glacial influence component *Glacier* vs. two other environmental components (*Instream* and *Resources*; see Environmental components section) in structuring benthic metacommunity.

Model 2 compared the relative importance of the environmental glacial influence component *Glacier* vs. spatial variables to explain metacommunity structure. Because spatial processes in aquatic networks potentially occur through overland, watercourse and directional downstream dispersal, we built three eigenfunction-based spatial variables (overland, watercourse and directional downstream; see Computation of eigenfunction-based spatial variables section). In addition, as both geographical and altitudinal distances might enhance community variation among sites [42–44] we assessed the relative contribution of the three spatial variables computed using either geographical or altitudinal distances. In this model, we thus tested whether both geographical and altitudinal distances acted as dispersal barriers for macroinvertebrates.

Model 3 assessed the effect of two spatial variables (watercourse and directional downstream) computed using glaciality distances (difference in glaciality among sites following the stream channel, see Glaciality distance section). Here, we tested whether the difference in glaciality among sites along the stream channel acted as an aquatic dispersal barrier for macroinvertebrates.

To assess the influence of taxa dispersal ability and site location on our predictions, we ran each of the three models using four taxa matrices (namely *All taxa*, *Flying taxa*, *Non-flying taxa*, and *First-order taxa)*. *All taxa* consisted of an abundance matrix including all identified taxa. *Flying taxa* consisted of an abundance matrix of taxa with a winged adult life stage while *Non-flying taxa* comprised exclusively aquatic taxa. *First-order taxa* matrix consisted in an abundance matrix of all taxa found in first-order sites. Due to the lack of data on the dispersal ability of Andean stream macroinvertebrate taxa, we were not able to construct a precise classification of dispersal abilities (e.g. [45]), and therefore divided taxa into only two groups (*Flying taxa* and *Non-flying taxa*). The four abundance matrices were transformed using Hellinger transformation prior to statistical analysis (see [46] for details) using the package *vegan* in R.

### Data preparation

#### Geographical distance

We calculated the following geographical distances: the overland distance, the shortest straight line distance between sites was calculated in ArcGis (version 10.0) using the *Analysis/Proximity/Point distance* tool; and the watercourse distance, the distance among sites following the stream channel was calculated after creating the stream network using the *Network Analyst* tools in ArcCatalog (version 10.0) and then computing the distance following the stream channel in ArcGis using both *Network Analyst/Make OD Cost Matrix* and *Add location* tools.

#### Altitudinal distance

We calculated overland and watercourse altitudinal distances between all pairs of sites as the elevational difference between the highest and the lowest point along the Euclidean line between sites, and the elevational difference between the highest and the lowest point along the stream channel, respectively. A digital elevation model (DEM) was created using a 40-m resolution contour line from the Ecuadorian Military Geography Institute (available at http://www.igm.gob.ec/site/index.php) in ArcGis. Then overland and watercourse features between all pairs of sites were created in ArcGis using the *Linear referencing/Create routes* tool, and the features were attributed with elevation information derived from the DEM using the *3DAnayst/Functional surface/Add surface information* tool.

#### Glaciality distance

We calculated the difference in glacial influence among sites using the glaciality index provided by Ilg and Castella [47]. At each sites, water temperature, conductivity, 1/turbidity (≈1/suspended sediment) and 1/Pfankuch were scaled between 0 and 1, processed using a non-centred principal component analysis (NPCA) performed in R using the package *vegan*. Ordination scores of the sites along the first axis were used as an index of glaciality. The first axis values were transformed to obtain only positive values, with the highest values corresponding to the most glacially-influenced sites. Note that contrary to Ilg and Castella [47], this index was also applied to non-glacial streams, which allowed assigning a quantified environmental stress to those streams. A glaciality value was then assigned to all stream segments; a segment is either a reach between two sites if there is no tributary between the two sites or a segment between a site and a tributary. In most cases, the glaciality value assigned to a segment was the glaciality value calculated at the corresponding upstream sites, otherwise the one calculated at the downstream sites (i.e. for the first segments downstream tributaries). Finally the difference between the highest and the lowest glaciality was calculated among all pairs of sites following the stream channel.

#### Environmental components

We constructed three environmental matrices: the *Glacier* matrix composed of temperature, conductivity, 1/turbidity and 1/Pfankuch, the *Instream* matrix composed of stream width, water depth, slope, and pH, and the *Resources* matrix composed of epilithic algae and benthic organic matter. All variables were previously scaled between 0 and 1.

### Data analysis

#### Computation of eigenfunction-based spatial variables

Eigenfunction-based spatial variables (eigenvectors) were generated from overland distance and watercourse distance matrices using Moran’s Eigenvector Map (MEM) analysis [48], and from a directional downstream distance matrix using Asymmetric Eigenvector Map (AEM) analysis [49]. More details on the methods are given in S2 Appendix. In exactly the same way, spatial eigenvectors were computed using either geographical, altitudinal, or glaciality distances. Then, for each distance (e.g. overland geographical distance), a global test was performed using all spatial eigenvectors with positive eigenvalues in a redundancy analysis. When global tests were significant, we proceeded with a forward selection procedure to reduce the number of spatial eigenvectors to make the model more parsimonious (see [50] for details). Those preliminary analyses were performed in R using the package *packfor* and *vegan*. Note that we did not compute the eigenvectors from overland glaciality distances as it was not relevant; there is no difference in glaciality among sites through the air.

#### Community variation partitioning

Variation partitioning was performed using redundancy analysis ordination to assess the relative performance of the different explanatory variables on the structure of species communities in our three models [51]. It quantifies the percentage of community variation explained exclusively by each explanatory variable (unique contribution), as well as the shared variance explained by various explanatory variables (confounded effects between various, and sometimes interrelated explanatory variables). The significance of each unique fraction was tested with 999 permutations [52, 53]. Those analyses were performed using the package *vegan* in R.

In addition, we also tested whether the effects of geographical, altitudinal and glaciality distances were not confounded. For this, variation partitioning analyses were separately performed on spatial eigenvectors overland, watercourse and directional downstream using geographical, altitudinal and glaciality distances. All those analyses were performed for the four taxon matrices.

## Results

First-order glacier-fed and groundwater streams presented high and low glaciality values, respectively. Glaciality values in glacier-fed streams decreased downstream with increasing contribution in groundwater. We identified a total of 85 taxa from the 51 sites. Local richness ranged from 2 (at site 3) to 37 taxa per site (at site 28, Fig 1A) and macroinvertebrate density ranged from 176 (at site 3) to 42 256 ind.m^-2^ (at site 35). Fourteen taxa occurred only in glacier-fed streams, among which six exclusively in first-order. Fifteen taxa occurred only in groundwater streams, among which eleven exclusively in first-order. Dominant taxa were Orthocladiinae, *Podonominae* sp1, and *Simulium* in first-order glacier-fed streams; Orthocladiinae, *Andesiops*, and *Alluaudomyia* in main glacier-fed streams; and Orthocladiinae, *Hyallela*, and *Andesiops* in groundwater streams. Among the 85 taxa identified at the whole catchment scale, we found 72 and 68 taxa in first-order and main streams sites, respectively. Seventy taxa had a winged adult life stage while only 15 were exclusively aquatic taxa. Flying and non-flying taxa corresponded to 76 and 24% of the total abundance, respectively. Among the 15 exclusively aquatic taxa, only six occurred in first-order glacier-fed streams among which four in very low number (< 7 individuals).

### Glacier among environmental components (model 1)

**Fig 2:**
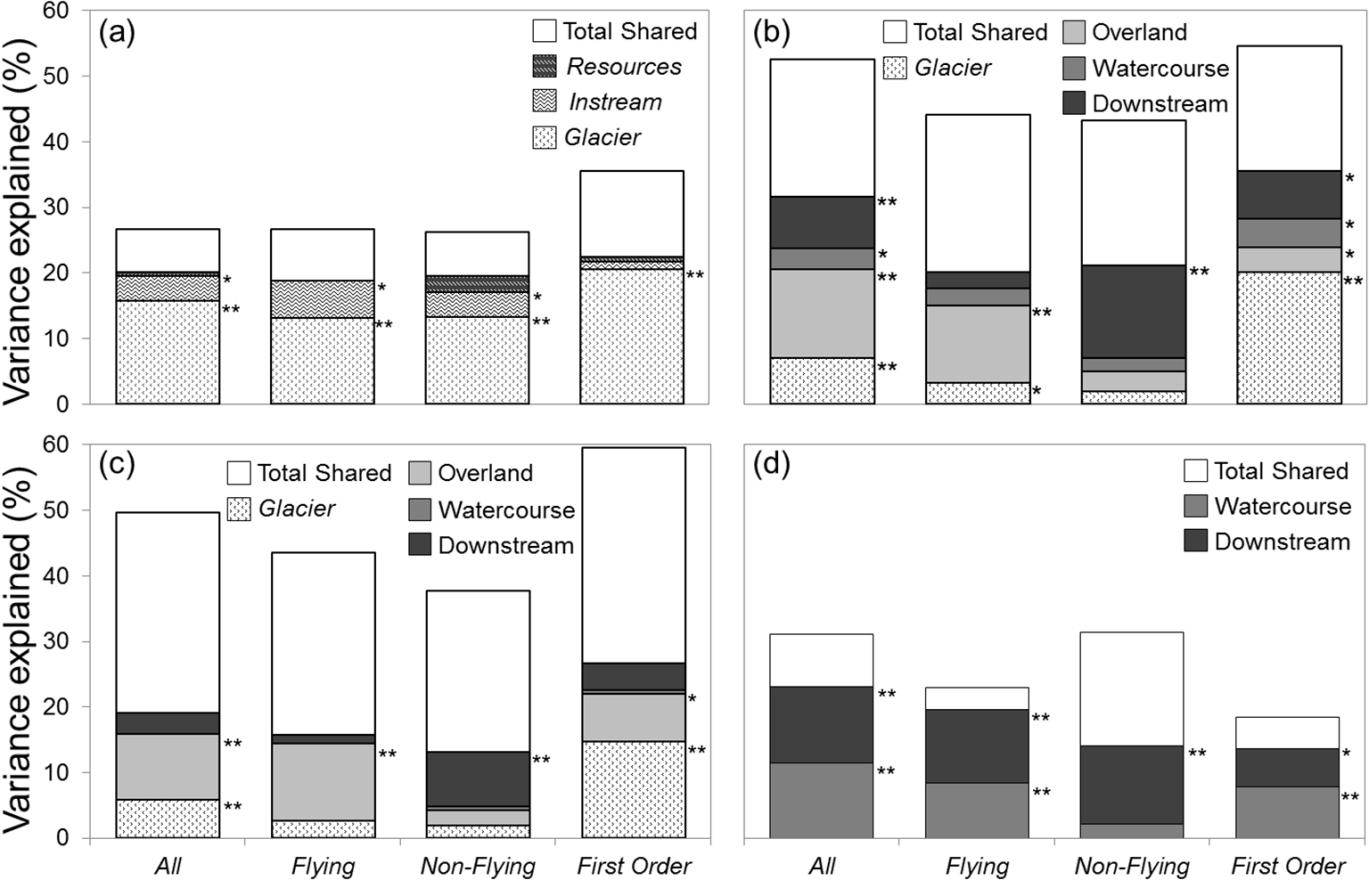
Results of variation partitioning analyses. Results of analyses performed on (**a**) the two environmental components *Instream* and *Resources* and the component *Glacier*, (**b**) the three spatial eigenfunction-based variables computed using overland, watercourse, downstream geographical distances and the environmental component *Glacier*, (**c**) the three spatial eigenfunction-based variables computed using overland, watercourse, downstream altitudinal distances and the environmental component *Glacier*, and (**d**) the two spatial eigenfunction-based variables based on watercourse and downstream glaciality distances. All analyses were performed for the four taxon matrices (*All taxa*, *Flying taxa*, *Non-flying taxa*, and *First-order taxa*). Figure shows the amount of variation (%) in the structure community that is uniquely explained by each explanatory variable as well as the total shared variance (all the shared components between two, three and four explanatory variables). The level of significance is indicated next to the bars (** P < 0.01, * P < 0.05, see S3 Appendix for details).

In total, we found that the three environmental components *Glacier*, *Instream*, and *Resources* explained between 26.1 and 35.6% of the community variation depending on the taxon matrix considered, among which *Glacier* always had the largest contribution (Fig 2A, details on the fractions of explained variances are given in S3 Appendix). The total portion of community variation explained by all environmental components, as well as the unique contribution of *Glacier*, were larger for first-order sites (35.6 and 20.6%, respectively) than when considering all sites together (26.2 and 15.7%, respectively). *Instream* had a significant (but low) unique contribution (around 4%) for *All taxa*, *Flying taxa* and *Non-Flying taxa*, while *Resources* had no significant effect on community variation.

### Spatial variables and Glacier (models 2 and 3)

The three spatial variables (overland, watercourse and downstream) computed with geographical distances and *Glacier* contributed almost half of community variation (residuals < 60%, Fig 2B). However, the relative unique effect of spatial variables and *Glacier* on community variation differed considerably among taxon matrices. Total unique contribution of spatial variables was larger than the unique contribution of *Glacier* for *All taxa* (27.2 against 7%), while it was the opposite for *First-order taxa* (16.1 vs. 20.2%). For *All taxa* and *Flying taxa*, overland variables best explained community variation (13.6 and 11.9%), while downstream variables did for *First-order taxa* and *Non-Flying taxa* (7.2 and 14.1%). When computing the three spatial variables (overland, watercourse and downstream) using altitudinal distances (Fig 2C), we found that overland variables best explained community variation of *All Taxa*, *Flying Taxa*, and *First-order taxa* while downstream variables did so for *Non-Flying taxa*.

Both watercourse and downstream variables computed with glaciality distances (model 3) explained between 18.5 and 31.5% of the community variation depending on taxa matrix (Fig 2D). Both watercourse and downstream variables significantly explained community variation of *All taxa*, *Flying taxa* and *First-order taxa*, while only downstream variables did so for *Non-flying taxa*.

**Fig 3:**
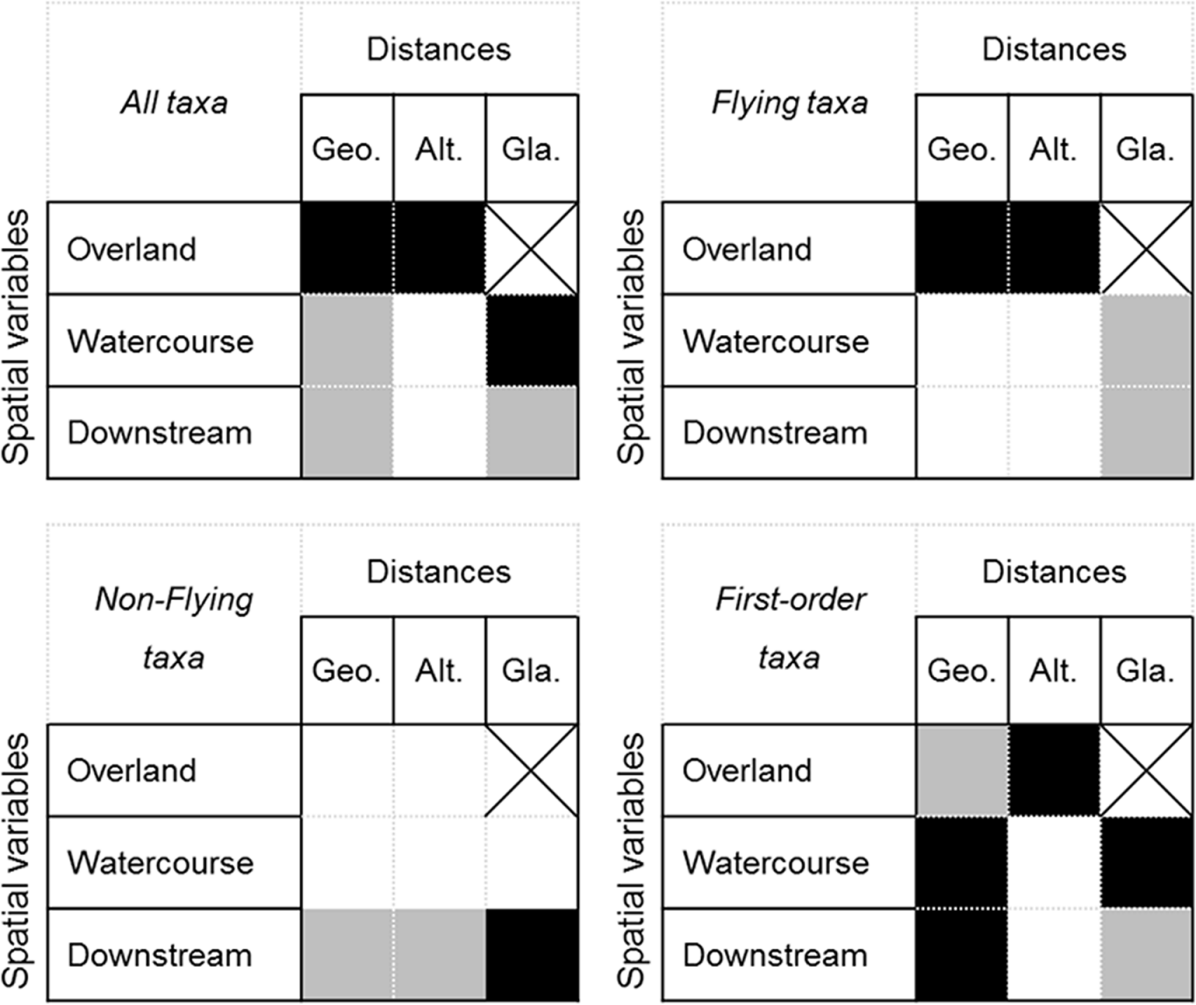
Summary of the results of variation partitioning analyses. Illustrative figure indicating, for each spatial variable (overland, watercourse, and downstream) which distance type (geographical, Geo., altitudinal, Alt., and glaciality, Gla.) had a significant effect on community variation of *All taxa*, *Flying taxa*, *Non-flying taxa*, and *First-order taxa*. A black mark indicates that the corresponding distance had a unique significant effect on community variation, and that its effect was not confounded with others types of distance. A grey mark indicates that the corresponding distance had a unique significant effect on community variation, but that its effect was confounded with at least one of the others type of distance.

For a given spatial variable, effects of geographical, altitudinal and glaciality distances on community variation were often highly confounded (see S4 Appendix). For example, the effect of downstream variables based on geographical distances on community variation of *Non-flying taxa* was highly confounded with the effects of downstream variables based on altitudinal and glaciality distances (see S4 Appendix). Taking into account these confounding effects allowed us to determine which distance type had a unique significant effect on community variation (Fig 3). Overland geographical and altitudinal variables as well as the glaciality watercourse variables had significant unique contributions to community variation of *All taxa*. Overland geographical and altitudinal variables had a significant unique contribution to community variation of *Flying taxa* and downstream glaciality variables of *Non-flying taxa*. Watercourse and downstream geographical variables, altitudinal overland, and glaciality watercourse variables had a significant unique contribution to community variation of *First-order taxa*.

## Discussion

### Mechanisms driving the metacommunity structure in a glacial stream network

The use of metacommunity theory as a central conceptual framework allowed us to identify mechanisms driving the benthic macroinvertebrate metacommunity structure in a glacial stream network. In agreement with most previous studies (e.g., [47, 54, 55]), we found that environmental filtering was an important mechanism structuring benthic communities in glacier-fed streams, and that glacial influence was the primary environmental filter. At the whole catchment scale, we found that both environmental and spatial variables significantly contributed to community variation, suggesting that both dispersal processes and environmental filtering drove metacommunity structure [56, 57], a pattern supported by various recent studies on macroinvertebrate metacommunities in non-glacial stream networks (e.g., [58–62]). However, clear differences were observed between hierarchical scales in the catchment (whole catchment vs. first-order sites). While environmental filtering better explained community variation than dispersal processes for first-order sites, it was the contrary at the whole catchment scale. This result is in line with the study of Brown and Swan [26] who suggested that headwater macroinvertebrate communities are structured according to a species sorting paradigm (moderate dispersal allowing species to sort along environmental gradients) due to their isolation from the regional species pool [31], while mainstem communities are structured according to a mass effects paradigm (high rates of dispersal interacting with local influence, regional processes dominate [63]).

Overland spatial variables based on geographical distances best explained community variation, especially for taxa with flying stage. This indicates that aerial dispersal was a major dispersal pathway in our studied stream network, which has been already reported in both non-glacial [64–66] and glacial stream networks [55]. Directional downstream spatial variables based on geographical distances also contributed in a significant way to community variation, especially for exclusively aquatic taxa, although geographical, altitudinal and glaciality distance effects were confounded. This suggests that downstream drift was also a major dispersal pathway in our studied watershed, which was not surprising as glacial floods occurred regularly [35, 67] and generated a daily increase in the downstream water flux. This result is supported by Jacobsen et al. [68] who, in the same study area, showed elevated macroinvertebrate drift and downstream displacement of fauna during glacial floods. This pattern was also evidenced in a fishless alpine glacier-fed stream by Robinson et al. [69] who found the diel drift pattern was associated with the summer afternoon peak in discharge. In agreement with those results, Robinson et al. [70] and Zbinden et al. [71] identified macroinvertebrate drift as an important colonization pathway in glacier-fed streams.

Overland spatial variables based on altitudinal distances significantly explained community variation, especially for flying taxa. While altitude is often used as a proxy of other environmental variables (e.g., temperature, vegetation type; [57]) to explain community variation among sites, altitudinal distances are often neglected in freshwater metacommunity studies (e.g., [21, 30]). By contrast, our study pointed out that altitudinal distances could be an important component of macroinvertebrate dispersal limitation in alpine stream network (see also [42, 72]).

In addition, we found that directional downstream spatial variables based on glaciality distances had a significant unique contribution to community variation of exclusively aquatic taxa. This result suggests that directional downstream dispersal of non-flying taxa between neighboring sites was limited by the difference in glaciality among those sites. As the abundance of non-flying taxa was very low in first-order glacier-fed streams, difference in glaciality likely reduced macroinvertebrate drift between neighboring groundwater and main glacier-fed sites (see also [73, 74]). Moreover, we found that aquatic non-directional dispersal was limited by the difference in glaciality among sites. As not all macroinvertebrates are adapted to environmental harshness of glacial meltwater [33, 75] aquatic non-directional displacement through glacier-fed streams was probably restricted to a few taxa such as cold stenotherms [55]. Consequently, glacier runoff likely acts as a dispersal barrier, isolates headwater streams, and restricts the colonization throughout the catchment of species not adapted to harsh glacial conditions.

While glaciality appeared to be a key driver of macroinvertebrate metacommunity structure, part of it remained unexplained as reported in most stream metacommunity studies [76]. This might partly be due to unmeasured environmental variables such as oxygen availability [77, 78] and discharge or current velocity [79, 80], all known to affect macroinvertebrate distributions. Unexplained variance could also be due to unstudied species interactions, another local process that might affect metacommunity structure [21, 81]. Species interactions include interactions among species from the same trophic level (e.g., facilitation, competition; [82]) but also among species from various trophic levels along the food web (e.g., herbivory, predation; [64, 83]). Indeed, although we found epilithic algae and benthic organic matter had no significant effect on community variation, other potential food sources such as rooted submerged macrophytes (mainly *Myriophyllum* and *Callitriche*), mosses, and filamentous algae (*Microspora* and *Vaucheria*) present in our study area might contribute to the spatial variability in macroinvertebrate communities [33, 84]. Likewise, rainbow trout (*Oncorhynchus mykiss*, even present in glacier-fed streams; [85]) might also affect macroinvertebrate spatial organization [86]. Also, a better knowledge of dispersal abilities and strength of benthic species (e.g., adult flight, drifting propensity, swimming and crawling strength; [45]) would allow better understanding metacommunity dynamics [87, 88]. For example, poorly-dispersing organisms may show stronger spatial structuring and weaker environmental control of community structure than stronger dispersers [29, 89], as low dispersal rates (i.e. dispersal limitation) prevent species to reach environmentally suitable sites. However, at small spatial scales, strong dispersers should also show low environmental filtering as very high dispersal rates (i.e. mass effect) homogenize community structure at adjacent sites independently of their environmental conditions [90]. Moreover, adult flight of some taxa tends to be concentrated along riverine corridors [64, 91, 92] implying that watercourse spatial variables might also reflect aerial dispersal along streams for some taxa. Finally, part of the unexplained variance could also reflect the high level of context dependency emerging in metacommunity patterns [76, 93].

### Predicting the fate of aquatic macroinvertebrate diversity throughout the glacier retreat process

**Fig 4:**
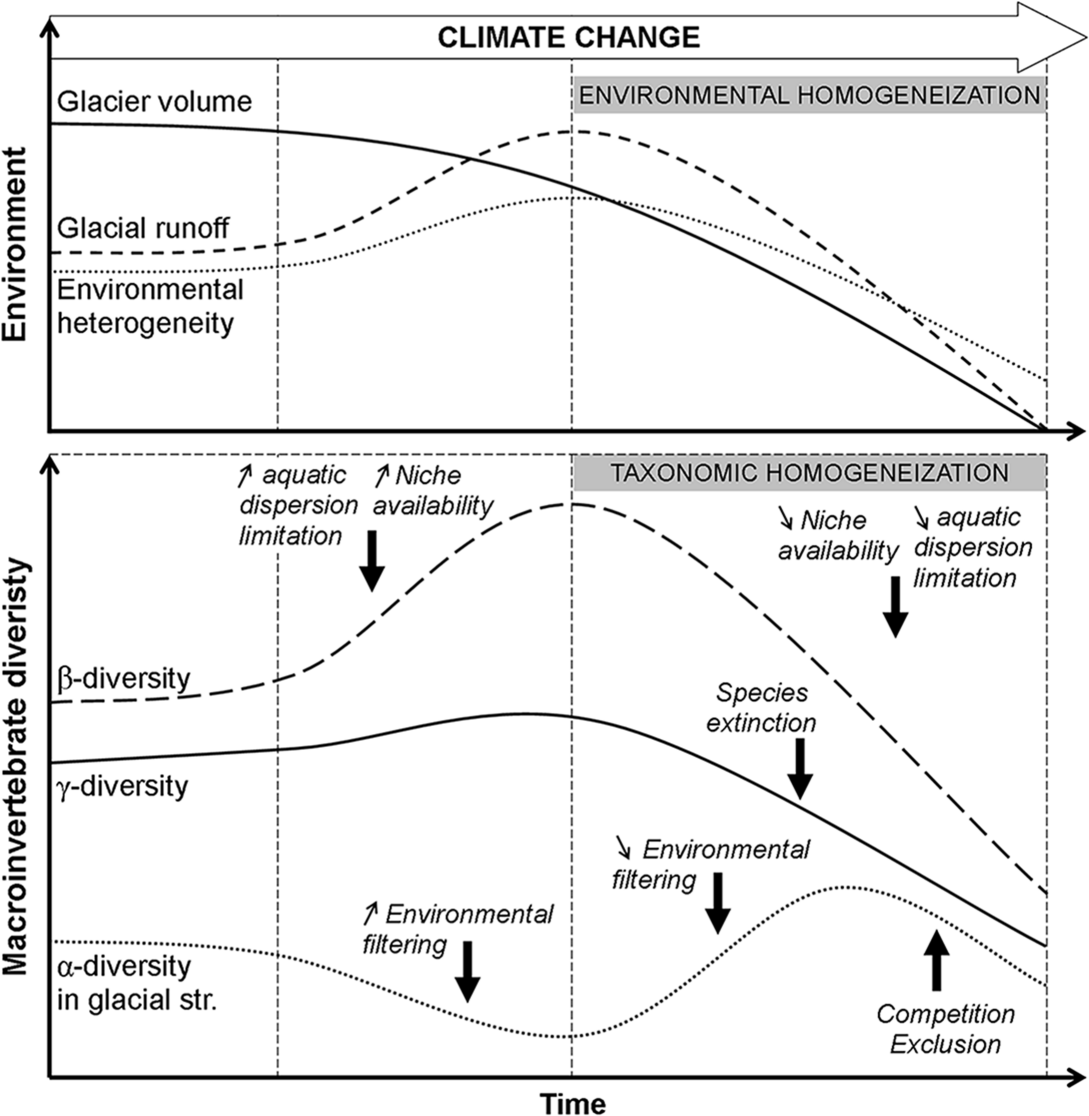
Conceptual diagram illustrating expected temporal changes in alpine aquatic diversity under the ongoing climate change: α-diversity in glacier-fed streams, β-diversity, and γ-diversity in glacierized catchments according to alteration in glacial meltwater contribution to alpine streams flow. This conceptual diagram was built based on the mechanisms structuring the aquatic macroinvertebrate metacommunity revealed in this study and those from the literature on the organization of α, β, and γ diversity. Species extinction included species in headwater streams (both glacier-fed and groundwater streams). Up arrow and down arrow symbols signified increase and decrease, respectively.

Based on the metacommunity processes identified in this study and on mechanisms proposed in the literature to explain the organization of local (α), among sites (β), and regional (γ) diversity in glacierized catchments, we propose a conceptual framework of macroinvertebrate diversity response to glacier runoff alteration under the ongoing climate change (Fig 4).

Under a scenario of increase in glacier runoff, we first assume an increase in glaciality, in environmental harshness [47, 94], and consequently an increase in environmental filtering. As α-diversity is typically very low in glacier-fed streams due to the harsh glacial conditions allowing only adapted species to survive [33, 95], we expect a decrease in α-diversity in glacier-fed streams. Second, we anticipate an increase in environmental heterogeneity within catchments and consequently an increase in glaciality distances among sites. In agreement with previous studies, our results showed that this high heterogeneity generates high community variation among sites [11, 96, 97]. We thus expect an increase in β-diversity under this increase in habitat types, i.e. in niche availability. Moreover, our results showed that glacier runoff not only acts as an environmental filter, but also limits macroinvertebrate dispersal, thereby restricting both species establishment and waterborne dispersal (both directional and non-directional). Hence, any increase in glacier runoff should limit even more the colonization and establishment of generalist species not adapted to the harsh glacial conditions, and consequently, enhance β-diversity [94, 98].

As opposed to this, we expect that any reduction in glacier runoff should lead to a decrease in environmental harshness and consequently to a reduction in environmental filtering, allowing more species to establish, thereby increasing α-diversity in glacier-fed streams [11]. However, previous studies evidenced a hump-shaped relationship between glacial influence and α-diversity, suggesting competition exclusion by high dominance of competitively superior species at low level of glacial influence, and environmental filtering at a high level of glacial influence [13, 97]. We thus assume that α-diversity in glacier-fed streams would increase as environmental filtering decreases until an intermediate level of glacier runoff alteration, and then decrease until the complete disappearance of the glacier due to competition exclusion at low level of glacial influence. We also expect an environmental homogenization, leading to change in metacommunity structure and dynamics. Indeed, although macroinvertebrate dispersal would still be limited by geographical and altitudinal distances in a catchment without glacier runoff, aquatic dispersal would not be limited anymore by glacial meltwater. Therefore, glacier runoff reduction should facilitate dispersion of generalist species not adapted to the harsh glacial conditions into new sites and promote their establishment. Reduction in both environmental filtering and dispersal limitation would thus diminish β-diversity [11, 13]. Moreover, in addition to being threatened by changes in environmental conditions [9, 99] specialized species (including endemic ones) in glacial and groundwater headwater streams (17 specialist taxa in our study stream network, i.e. 20% of the regional pool) are also threatened by the upward migration of potentially more competitive species [100, 101] as they will no longer be isolated by “inhospitable” stream conditions [102], i.e. glacial meltwater. Species survival would thereby strongly depend on species’ ability to shift to suitable habitats [103]. As macroinvertebrate dispersal is also limited by elevation, some species might not be able to reach upper suitable habitats before the colonization of generalist species. Therefore, under global warming, reduction in glacier runoff might induce both extinction of specialized species and a taxonomic homogenization within glacierized catchments, resulting in an irreversible reduction in regional diversity [11, 13].

## Supporting Information

S1 Appendix Illustration showing that temporal changes in community composition among various dates are significantly lower than the spatial changes in community composition among various stream sites in our study area. Mean values (95% CI) of the spatial and temporal macroinvertebrate community dissimilarities calculated for two sets of streams (set 1 and 2) based on Bray-Curtis index (Baselga 2010—*Global Ecol*. *Biogeogr*.). Set 1 includes three first-order stream sites along the same glacier-fed stream sampled 10 times within two years. Set 2 includes one first-order glacier-fed stream, one first-order groundwater stream, and one second order mixed stream sampled 16 times within two years. Temporal pairwise dissimilarities were calculated among all sampling dates for each stream site. Spatial pairwise dissimilarities were calculated (independently for set 1 and 2) among all stream sites for each sampling date. One-way ANOVA followed by Tukey tests were performed independently for set 1 and 2 to test whether spatial pairwise dissimilarity values were significantly different than temporal pairwise dissimilarity values. Mean values followed by different letters are significantly different (p-value < 0.01 for both sets, F = 122.42 and 43.89 for set 1 and 2, respectively; one way ANOVA).
(TIF)

S2 Appendix Details on methods: Computation of eigenfunction-based spatial variables. (DOCX)

S3 Appendix Details of each component (unique and shared) of the variation partitioning analyses performed for models 1, 2, and 3. Details of each component (unique and shared) of the variation partitioning analyses performed for *All taxa*, *Flying taxa*, *Non-flying taxa*, and *First-order taxa* on (1) the three environmental components (*Glacier*, *Instream* and *Resources*); (2) the three spatial variables overland, watercourse, downstream computed using geographical distances and the environmental component *Glacier*; (3) the three spatial variables overland, watercourse, downstream computed using altitudinal distances and the environmental component *Glacier*; and (4) the two spatial variables watercourse, downstream computed using glaciality distances. Upper numbers inside the circles represented the amount of variation that is uniquely explained by each explanatory variable and the shared part of variance explained between all pairwise variables. For geographical and altitudinal distances, lower bold numbers inside the circles represented the shared part of variance explained between each spatial variable and the environmental component *Glacier*. Number outside the circle corresponded to the total amount of community variation explained by each explanatory variable excluding the portion shared with the environmental component *Glacier*. For geographical and altitudinal distances, lower bold number outside the circles corresponded to the total amount of community variation explained by each explanatory variable including the portion shared with the environmental component *Glacier*. Residuals correspond to the percentage of the community variance unexplained by the model. For geographical and altitudinal distances panels, Gla corresponds to the unique portion explained by the environmental component *Glacier*, Tot Gla corresponds to the total fraction explained by the environmental component *Glacier* (including the spatially structured part). Tot Spa corresponds to the total fraction explained by the three spatial variables excluding the parts shared with the environmental component *Glacier*. The empty fractions correspond to explanatory variables that explain less of the community variation than would be expected by chance.
(TIF)

S4 Appendix Confounded effects of geographical, altitudinal, and glaciality distances. Results of variation partitioning analyses performed on the overland spatial eigenfunction-based variables computed using geographical (geo.) and altitudinal (alt.) distances at the top. Results of variation partitioning analyses performed on the watercourse spatial eigenfunction-based variables computed using geographical (geo.), altitudinal (alt.), and glaciality (gla.) distances on the bottom left. Results of variation partitioning analyses performed on the downstream spatial eigenfunction-based variables computed using geographical (geo.), altitudinal (alt.), and glaciality (gla.) distances on the bottom right. Analyses were performed for the four taxon matrices *All taxa* (**a**), *Flying taxa* (**b**), *Non-flying taxa* (**c**), and *First-order taxa* (**d**). The figure shows the amount of variation (%) in the structure community that is uniquely explained by each spatial variable as well as the shared portion. The level of significance was indicated next to the numbers (** P < 0.01, * P < 0.05). The empty fractions correspond to explanatory variables that explain less of the community variation than would be expected by chance.
(TIF)
