# Supplementary figures and images for: Invertebrate Metacommunity Structure and Dynamics in an Andean Glacial Stream Network Facing Climate Change
Source: PLoS One. 2015 Aug 26;10(8):e0136793. doi: 10.1371/journal.pone.0136793 (PMC4550352; doi:10.1371/journal.pone.0136793)

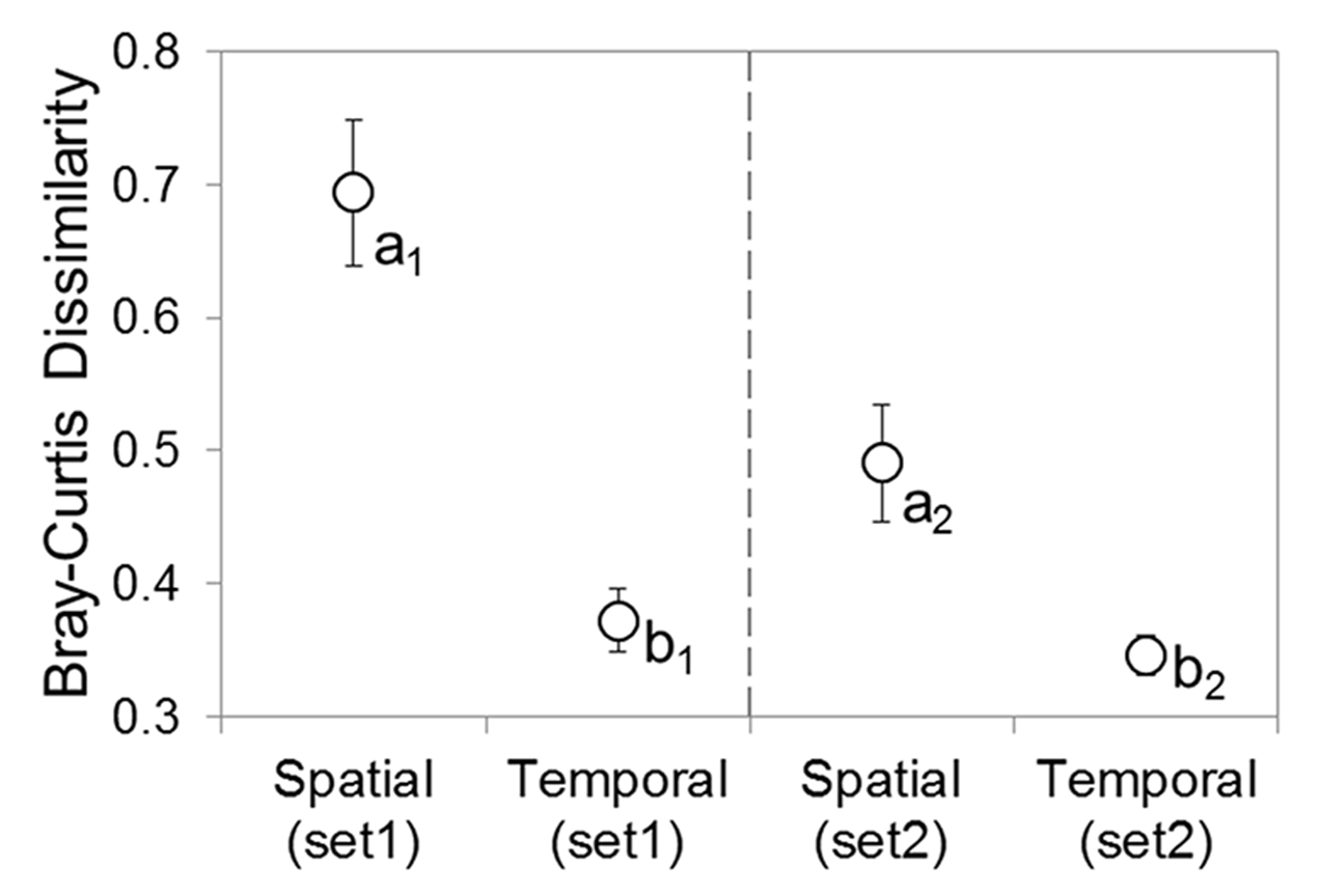

Supplement: S1 Appendix — Mean values (95% CI) of the spatial and temporal macroinvertebrate community dissimilarities calculated for two sets of streams (set 1 and 2) based on Bray-Curtis index (Baselga 2010—Global Ecol. Biogeogr.). Set 1 includes three first-order stream sites along the same glacier-fed stream sampled 10 times within two years. Set 2 includes one first-order glacier-fed stream, one first-order groundwater stream, and one second order mixed stream sampled 16 times within two years. Temporal pairwise dissimilarities were calculated among all sampling dates for each stream site. Spatial pairwise dissimilarities were calculated (independently for set 1 and 2) among all stream sites for each sampling date. One-way ANOVA followed by Tukey tests were performed independently for set 1 and 2 to test whether spatial pairwise dissimilarity values were significantly different than temporal pairwise dissimilarity values. Mean values followed by different letters are significantly different (p-value < 0.01 for both sets, F = 122.42 and 43.89 for set 1 and 2, respectively; one way ANOVA). (TIF) [file pone.0136793.s001.tif]

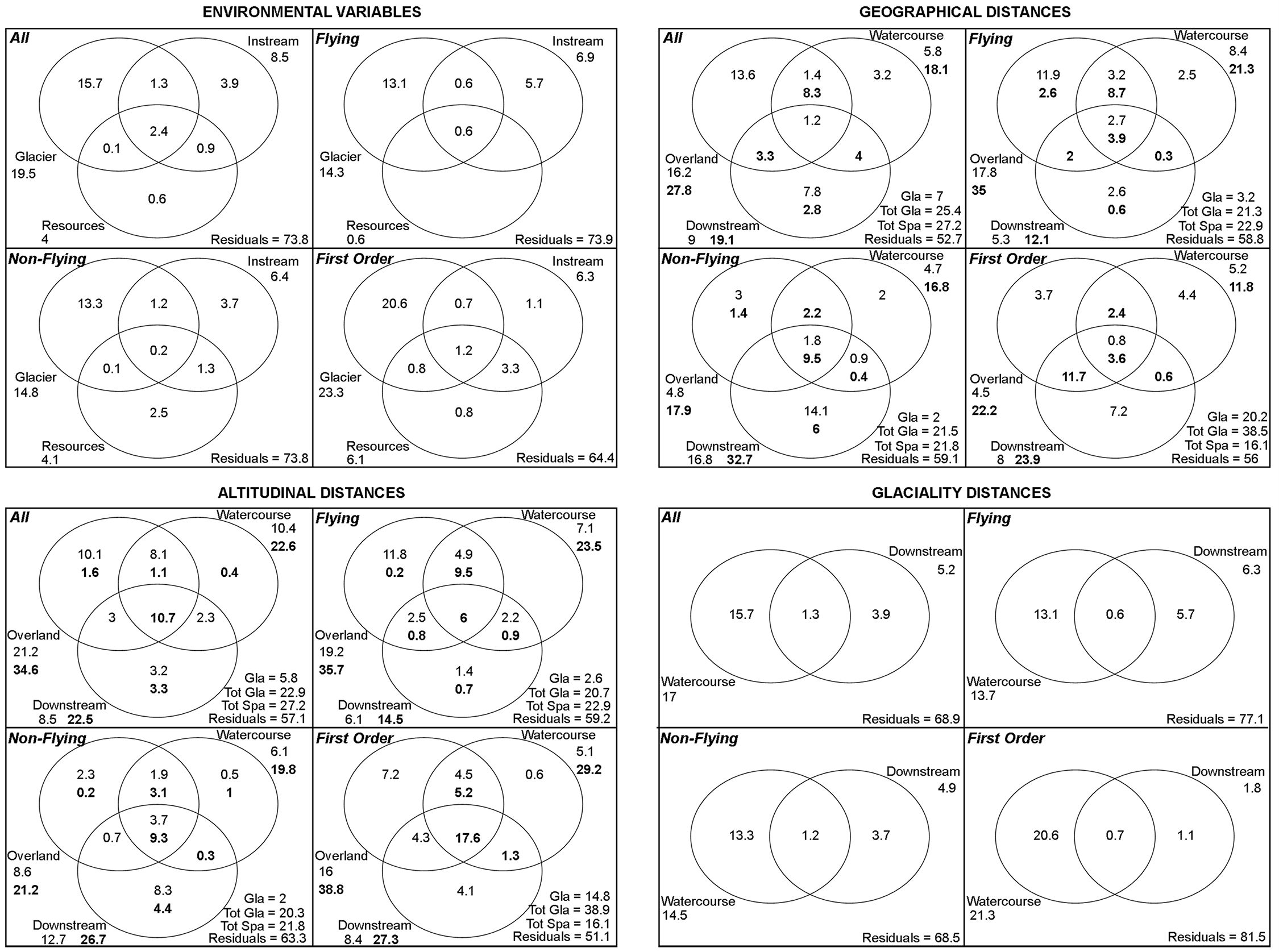

Supplement: S3 Appendix — Details of each component (unique and shared) of the variation partitioning analyses performed for All taxa, Flying taxa, Non-flying taxa, and First-order taxa on (1) the three environmental components (Glacier, Instream and Resources); (2) the three spatial variables overland, watercourse, downstream computed using geographical distances and the environmental component Glacier; (3) the three spatial variables overland, watercourse, downstream computed using altitudinal distances and the environmental component Glacier; and (4) the two spatial variables watercourse, downstream computed using glaciality distances. Upper numbers inside the circles represented the amount of variation that is uniquely explained by each explanatory variable and the shared part of variance explained between all pairwise variables. For geographical and altitudinal distances, lower bold numbers inside the circles represented the shared part of variance explained between each spatial variable and the environmental component Glacier. Number outside the circle corresponded to the total amount of community variation explained by each explanatory variable excluding the portion shared with the environmental component Glacier. For geographical and altitudinal distances, lower bold number outside the circles corresponded to the total amount of community variation explained by each explanatory variable including the portion shared with the environmental component Glacier. Residuals correspond to the percentage of the community variance unexplained by the model. For geographical and altitudinal distances panels, Gla corresponds to the unique portion explained by the environmental component Glacier, Tot Gla corresponds to the total fraction explained by the environmental component Glacier (including the spatially structured part). Tot Spa corresponds to the total fraction explained by the three spatial variables excluding the parts shared with the environmental component Glacier. The empty fractions c [file pone.0136793.s003.tif]

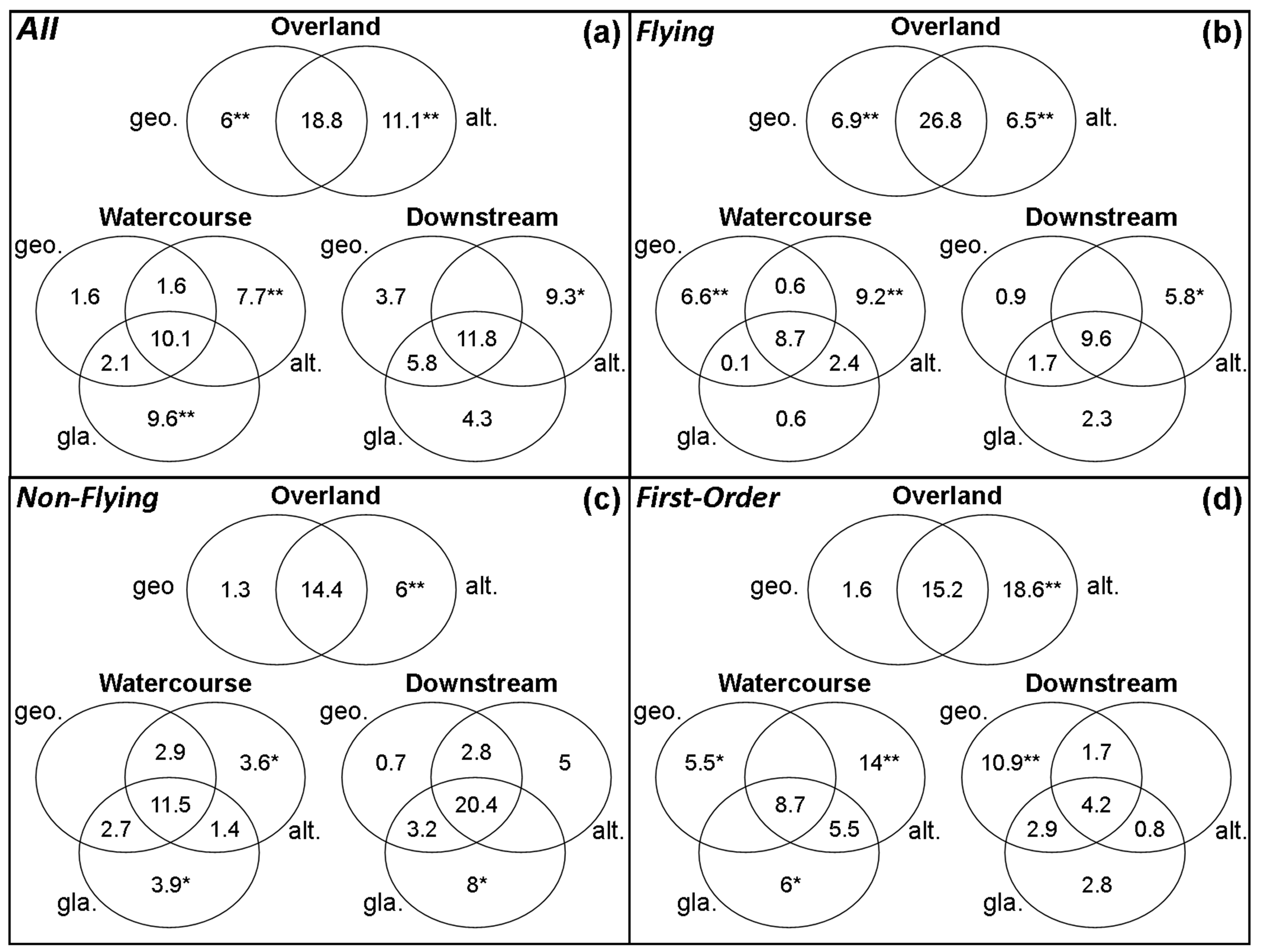

Supplement: S4 Appendix — Results of variation partitioning analyses performed on the overland spatial eigenfunction-based variables computed using geographical (geo.) and altitudinal (alt.) distances at the top. Results of variation partitioning analyses performed on the watercourse spatial eigenfunction-based variables computed using geographical (geo.), altitudinal (alt.), and glaciality (gla.) distances on the bottom left. Results of variation partitioning analyses performed on the downstream spatial eigenfunction-based variables computed using geographical (geo.), altitudinal (alt.), and glaciality (gla.) distances on the bottom right. Analyses were performed for the four taxon matrices All taxa (a), Flying taxa (b), Non-flying taxa (c), and First-order taxa (d). The figure shows the amount of variation (%) in the structure community that is uniquely explained by each spatial variable as well as the shared portion. The level of significance was indicated next to the numbers (** P < 0.01, * P < 0.05). The empty fractions correspond to explanatory variables that explain less of the community variation than would be expected by chance. (TIF) [file pone.0136793.s004.tif]
