# Supplementary material for: Invertebrate Metacommunity Structure and Dynamics in an Andean Glacial Stream Network Facing Climate Change
Source: PLoS One. 2015 Aug 26;10(8):e0136793. doi: 10.1371/journal.pone.0136793 (PMC4550352; doi:10.1371/journal.pone.0136793)
Supplement: S2 Appendix — (DOCX) [file pone.0136793.s002.docx]

**Appendix S2:** **Details on methods: Computation of eigenfunction-based spatial variables**

The computation of Moran’s Eigenvector Map (MEM) requires a connectivity matrix (i.e. the list of links among sites), and a matrix of weights to be applied to these links. The connectivity matrix is a binary matrix defining which pairs of sites are connected “1” and which are not “0”. The weighting matrix provides the intensity of the connections; the commonly used weighting function is: *weight = 1 - (d/d_max_)^3^*, where *d* is the distance between connected sites and *d_max_* the largest distance in the stream network. The final spatial weighting matrix results from the Hadamard (i.e. term- by term) product of these two matrices [1]. In our case, the connectivity matrix was constructed on the basis of the distance; i.e. a distance threshold was selected, and all sites within that distance were connected. 10 connectivity matrices were created with different thresholds: from the smallest distance connecting all sites (i.e. all sites are at least connected to one site) to the maximum distance found in the stream network (i.e. all sites are connected to the others 50 sites; Fig. S2). 10 spatial weighting matrices were constructed based on those 10 different connectivity matrices, but using the same weighting function. From each of the 10 spatial weighting matrices, MEM eigenvectors were computed and only the eigenvectors with positive eigenvalues were kept (i.e. corresponding to positive spatial correlation; [1, 2]). The contribution of each eigenvectors matrix (10 matrices) to the community variation was then tested. For this, the corrected Akaike Information Criterion (AICc) was computed for each eigenvectors matrix, and the spatial weighting matrix yielding the lowest AICc was retained. For example, the MEM eigenvectors, based on overland geographical distance, yielding to the lowest AICc for *All Taxa* matrix was computed for a distance threshold of 3222 m (Fig. S2). Finally, MEM eigenvectors were computed based on the spatial weighting matrix selected and only the eigenvectors with positive eigenvalues were kept. All analyses were performed in R using the package *spacemakeR* and *vegan*.

The computation of Asymmetric Eigenvector Map (AEM) requires information about the direction of the physical process, plus the same information as MEM (i.e. connectivity and weight). First the stream network was divided into edges (i.e. segments between two sites or between one site and one node). Then a rectangular sites-by-edges matrix E was constructed, including both the information of connection among sites and flow direction; *E_ij_ = 1* when the site *i* is influenced by the edges *j*; otherwise and *E_ij_ = 0* (see [3, 1] for sites-by-edges matrix examples). In our specific case, the process influence was assumed to come from upstream. Thus, the “starting points” were all uppermost stream sites (e.g., sites 3, 5, 25, see Fig. 1a). Then weight was assigned to the edge matrix based on the watercourse distance transformed by the same weighting function as the one used for the MEM eigenvectors. Those analyses were performed using the package *AEM* in R.

Note that when computing the watercourse connectivity matrices and the sites-by-edges matrices, we intentionally did not connect sites 1, 2, and 3 to any other one because the corresponding stream flowed to the north side of the volcano whereas the others streams flowed to the south side (connection among the streams did not occur before more than 350 km). Moreover, we did not connect sites 26, 27, 34, and 35 to the others sites due to the presence of a lake (Fig. 1a).

***
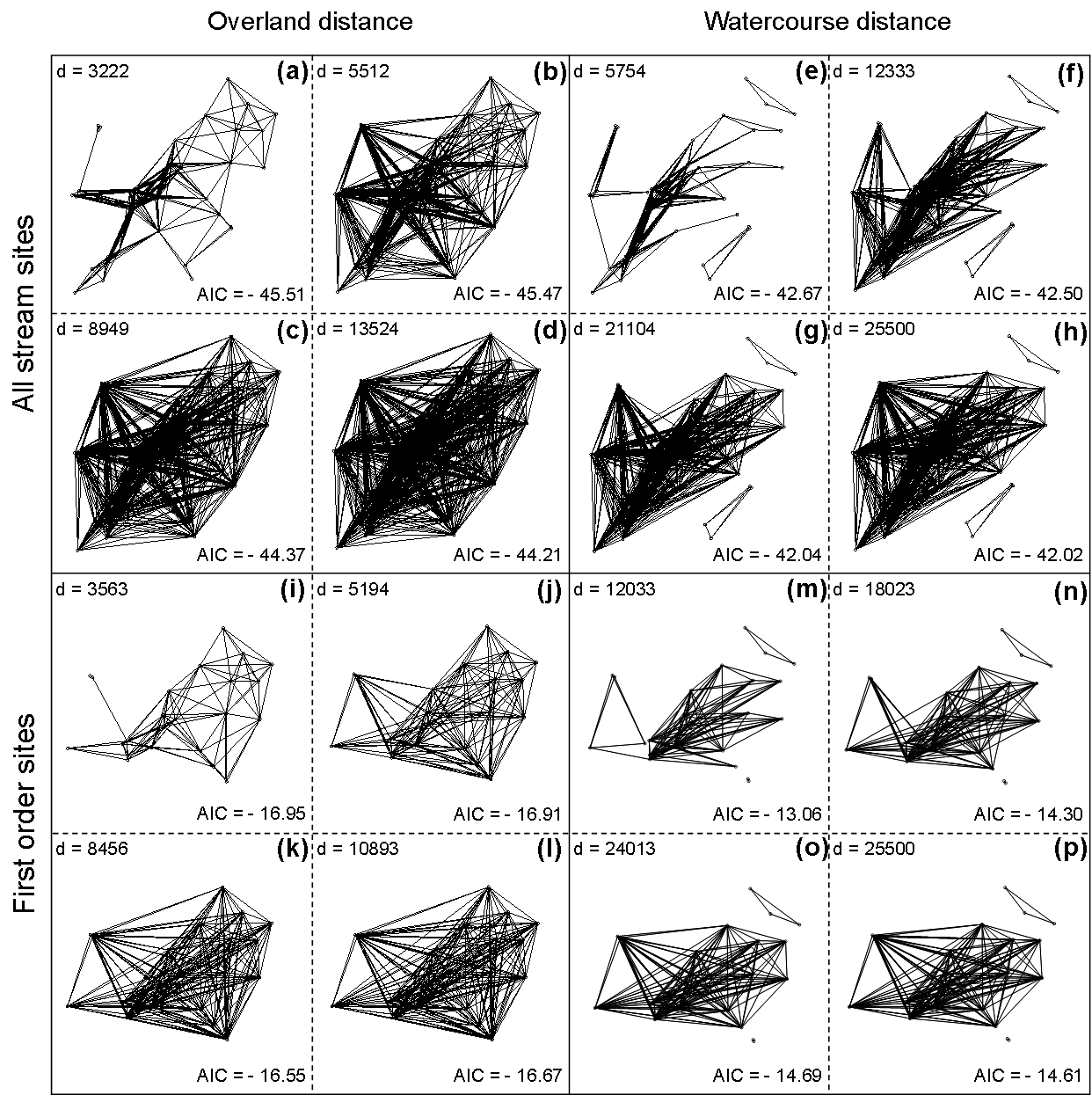
***

**Figure S2: Connectivity matrices.** Examples of connectivity matrices created based on different distance thresholds: from the smallest distance connecting all sites (i.e. all sites are at least connected to one site; **a**, **e**, **i**, and **m**) to the maximum distance found in the stream network (i.e. all sites are connected to the others 50 sites). Connectivity matrices were represented for overland (**a-d** and **i-l**) and watercourse (**e-h** and **m-p**) geographical distances for all study sites (**a-h**) and for first-order sites (**i-p**). Thresholds distance (d) and corresponding AIC_c_ were represented for each connectivity matrix. Note that in the connectivity matrices based on watercourse distance, six study sites were not connected to the other ones (see methods for justification).

**References:**

1. Borcard D, Gillet F, Legendre P. Numerical ecology with R: Springer; 2011.

2. Dray S, Legendre P, Peres-Neto PR. Spatial modelling: a comprehensive framework for principal coordinate analysis of neighbour matrices (PCNM). Ecol Model. 2006;196(3):483-493.

3. Blanchet FG, Legendre P, Borcard D. Modelling directional spatial processes in ecological data. Ecol model. 2008;215(4):325-336.
